# A Novel Hardware–Software Co-Design and Implementation of the HOG Algorithm

**DOI:** 10.3390/s20195655

**Published:** 2020-10-02

**Authors:** Sina Ghaffari, Parastoo Soleimani, Kin Fun Li, David W. Capson

**Affiliations:** Department of Electrical and Computer Engineering, University of Victoria, Victoria, BC V8W 2Y2 Canada; parastoo@uvic.ca (P.S.); kinli@uvic.ca (K.F.L.); capson@uvic.ca (D.W.C.)

**Keywords:** hardware–software co-design, histogram of oriented gradients, bin assignment, FPGA resource usage, accuracy loss, frame rate

## Abstract

The histogram of oriented gradients is a commonly used feature extraction algorithm in many applications. Hardware acceleration can boost the speed of this algorithm due to its large number of computations. We propose a hardware–software co-design of the histogram of oriented gradients and the subsequent support vector machine classifier, which can be used to process data from digital image sensors. Our main focus is to minimize the resource usage of the algorithm while maintaining its accuracy and speed. This design and implementation make four contributions. First, we allocate the computationally expensive steps of the algorithm, including gradient calculation, magnitude computation, bin assignment, normalization and classification, to hardware, and the less complex windowing step to software. Second, we introduce a logarithm-based bin assignment. Third, we use parallel computation and a time-sharing protocol to create a histogram in order to achieve the processing of one pixel per clock cycle after the initialization (setup time) of the pipeline, and produce valid results at each clock cycle afterwards. Finally, we use a simplified block normalization logic to reduce hardware resource usage while maintaining accuracy. Our design attains a frame rate of 115 frames per second on a Xilinx^®^ Kintex^®^ Ultrascale^™^ FPGA while using less hardware resources, and only losing accuracy marginally, in comparison with other existing work.

## 1. Introduction

Feature extraction is one of the main stages in a wide variety of pattern recognition applications [1,2,3]. In particular, feature extraction and description has been used in numerous computer vision algorithms for many applications. There have been many feature description algorithms proposed in the past decade, such as SIFT (scale-invariant feature transform) [3] and SURF (speeded up robust features) [4], which have all shown outstanding results in a wide variety of applications. HOG (histogram of oriented gradients) [5] is one of the commonly used descriptors which has proven to be useful in many computer vision applications, including human detection, car detection, and general object recognition.

An object detection system is typically a combination of an input sensor, a feature extraction module and a classifier to make decisions based on the extracted features. Since the main application of the HOG features is in human and object detection, the output of a camera sensor is given to the HOG descriptors, usually followed by a suitable classifier such as SVM (support vector machines). The main drawback of the HOG algorithm is its computational complexity, which prevents it from meeting the timing requirements of some practical applications. Therefore, many researchers have tried to implement this algorithm on hardware platforms such as GPUs (graphical processing units) and FPGAs (field programmable gate arrays) to reap the benefits from parallel computation and thus improve speed.

Ma et al. [6] compared the CPU (central processing unit), GPU, and FPGA implementation of the HOG algorithm. They implement the HOG algorithm on an Intel^®^ Xeon^®^ E5520 CPU processor, an Nvidia^®^ Tesla^®^ K20 GPU, and a Xilinx^®^ Virtex^®^-6 FPGA. Their FPGA implementation consumes 130× less energy than the CPU and 31x less energy than the GPU to process a single frame, while the speed is about 68x better than the CPU and 5x better than the GPU.

Since FPGA implementations typically consume less power than GPUs and CPUs, there has been considerable interest in FPGA implementation in numerous applications [7,8,9]. In particular, many scholars have contributed to FPGA implementation of the HOG algorithm [10,11,12,13,14]. Hardware implementations are usually evaluated by the four main metrics of speed, accuracy, power consumption and resource utilization. Since there are trade-offs between these metrics, many researchers aim to optimize a single metric depending on the specific application. One way to optimize these metrics is to benefit from the advantageous features of both hardware and software in implementing the algorithm. In these methods, the algorithm is partitioned into different functional stages and the most computationally complex stages are implemented on the FPGA. The stages that are sequential in nature or are controlling the data flow can be allocated to the CPU.

**Figure 1:**
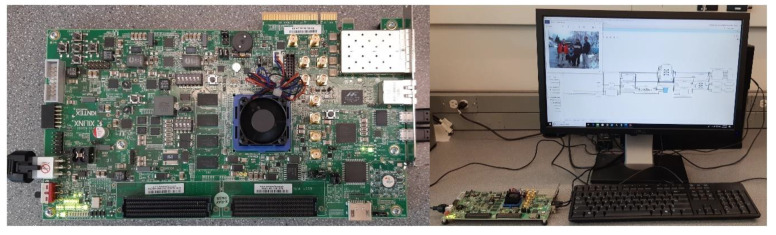
The KCU105 FPGA board (**left**) connected to the computer station (**right**) for experiments.

In this work, we propose a hardware–software co-design of the HOG algorithm. Our implementation consists of a fully pipelined HOG-SVM IP-core which is controlled by a MicroBlaze^™^ processor. MicroBlaze^™^ is a soft microprocessor core designed for Xilinx^®^ FPGAs. This design benefits from both the computational efficiency of the hardware and the simplicity of control mechanisms in software. Our method preserves accuracy and speed while decreasing resource utilization. Figure 1 shows the KCU105 FPGA board and the test environment of this research project. A sample image from the INRIA dataset [15] and the block diagram of the whole proposed system in Vivado^®^ software which we used are shown on the display. We used the UART (universal asynchronous receiver/transmitter) port to load the image in the system as a matter of experimental convenience. We could input the image in any other way and the results would be the same.

We have made four contributions in this paper. The first is an efficient task allocation between hardware and software. The second is a logarithm-based bin assignment in the HOG algorithm. The third is a hardware design for computing the histograms using two parallel modules. Finally, the fourth contribution is the approximation of the normalization level which preserves the accuracy of the system while reducing the hardware resource consumption.

In the rest of this paper, we introduce the HOG algorithm briefly in Section 2. Then, we review existing works that focus on hardware–software co-design of the HOG algorithm in Section 3. Then, we introduce our hardware–software co-design method in Section 4. In Section 5, we provide the details of our implementation. In Section 6, we compare the results of our design with other work and discuss the advantages and disadvantages of our design. Finally, in Section 7, we provide conclusions and future work directions.

## 2. Review of the Algorithm

In this section, we review the HOG algorithm and the SVM classifier briefly in Section 2.1 and Section 2.2, respectively.

### 2.1. The HOG Algorithm

**Figure 2:**
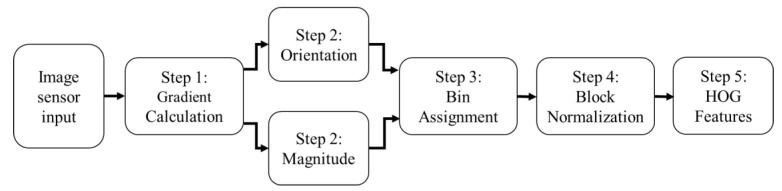
A flowchart of the HOG algorithm from input image sensor to HOG features.

The HOG algorithm has several steps, as shown in Figure 2.

In the first step, the derivatives in the horizontal and vertical directions are calculated for every pixel based on the adjacent pixels around them in a 3 by 3 neighborhood, as shown in Equations (1) and (2):

(1) $$G_{x}(x,y)=I(x+1,y)-I(x-1,y)$$

(2) $$G_{y}(x,y)=I(x,y+1)-I(x,y-1)$$

where I(x,y) represents the image pixel located in x and y coordinates, and $G_{x}$ and $G_{y}$ indicate the gradients of the horizontal and vertical directions, respectively.

In the second step, the magnitude of the gradients is computed as shown in Equations (3). In addition, the orientation of each pixel is calculated by computing the arctan value of the gradient in vertical direction $G_{y}$ over the gradient in the horizontal direction $G_{x}$, as shown in Equations (4).

(3) $$Mag(x,y)=\sqrt{G_{x}{}^{2}+G_{y}{}^{2}}$$

(4) $$\mathrm{Orientation}(x,y)=\tan^{-1}\frac{G_{y}}{G_{x}}$$

As shown in Figure 2, the next step adds the magnitude values to the bins according to the orientation of each pixel, for histogram generation.

In the fourth step, the histograms of the blocks are normalized separately. For block normalization, usually the L2-norm is used. For each block, which contains four histograms, the value of each bin in each histogram is multiplied by itself. The normalized value of each bin is the value of that bin divided by the square root of the summation of the squares of these values, as shown in Equations (5):

(5) $$h_{n}=\frac{h}{\sqrt{\sum {\left|h_{i}\right|}^{2}+\varepsilon }}$$

where $h_{n}$ is the normalized histogram, $h_{i}$ is the value of each bin, h is the initial histogram, and ԑ is a very small number to prevent division by zero. The final HOG features are the concatenation of the normalized histograms.

**Figure 3:**
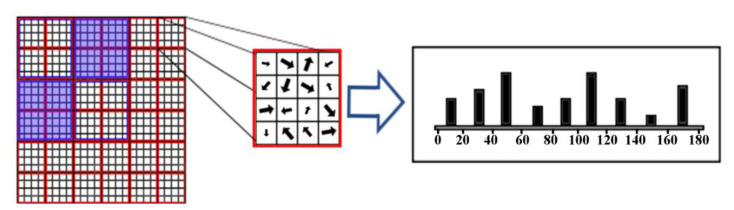
Visualization of cells (4 by 4 pixels) and blocks (each containing 4 cells) in the HOG algorithm.

HOG is computed for groups of pixels in the image. For example, every non-overlapping 16 pixels (4 × 4) form a cell and every four cells (2 × 2) form a block. Figure 3 represents this hierarchy. In Figure 3, the orientation of the gradient for each pixel is shown by arrows. The boldness and size of the arrows represent the magnitude of the gradients for that pixel.

### 2.2. Support Vector Machine

We chose an SVM as a classifier for two reasons. First, it is widely used with HOG features and has shown outstanding results, especially for human detection applications [5]. Second, the inference step of this classifier typically consumes fewer hardware resources than other classifiers, such as those based on neural networks. Therefore, after computing the HOG features, we use the SVM classifier for making decisions. An SVM is a linear classifier, which is used in many applications. In the training stage of the SVM classifier, the nearest samples to the decision boundary (support vectors) are determined. Using optimization techniques, this classifier maximizes the margin of the support vectors from the decision boundary.

In the testing phase, there is no optimization required. We can classify a sample using only precomputed weights of the SVM from the training stage and the feature vectors, as in Equations (6):

(6) $$f(x)=\sum w_{i}{}^{T}x+b$$

where x represents the input features, and $w_{i}$ and b are the weights and bias term learned by the classifier in the training stage, respectively. For classifying a sample, f(x) is compared to a threshold (normally zero) and a decision is made based on this comparison. Due to the accuracy and simplicity of the testing phase in an SVM classifier, it is a popular choice for hardware implementation.

## 3. Related Work on Hardware–Software Implementations

We have surveyed different methods for hardware implementation of the HOG algorithm, including an extensive review of methods with hardware–software co-design in our previous work [16,17]. In this section, first we briefly review the recent work implementing the HOG algorithm fully on hardware. Then, we review the work using hardware–software co-design methodology.

### 3.1. Hardware Implementation of the HOG Algorithm

There are several implementations of the HOG algorithm using pure hardware [11,12,13,14]. One of the benefits of implementing the HOG algorithm on hardware is of course speed enhancement. Implementing the whole algorithm on hardware is beneficial when resource consumption is not a constraint.

Qasaimeh et al. [12] propose a systolic architecture for hardware implementation of the HOG algorithm. They speed up the histogram generation by reusing the histogram bins generated for the adjacent cell. For each sliding window position, they subtract the contribution of the previous column of pixels from the histogram and add the contribution of the next column to the histogram to generate the new histogram value. They speed up their design using this method and achieve 48 fps for 1920 × 1080 images.

Long et al. [13] propose an ultra-high-speed implementation of the HOG algorithm for object detection. They use a high-speed vision platform which contains a high-speed camera FASTCAM SA-X2. The vision platform sends 64 pixels per clock cycle to the HOG computation module as input. Instead of storing the HOG values in a memory, they store only the maximum values of the HOG feature vector and its corresponding coordinates so as to simplify the computations of the further steps.

Ngo et al. [14] propose a long pipeline architecture for the HOG algorithm with 155 stages. Although their proposed system contains a processor and the FPGA part for the HOG algorithm, since they use the processor only for adding bounding boxes onto the output image we categorize this work as a hardware implementation of the HOG algorithm. In the HOG core, they use the CORDIC (coordinate rotation digital computer) algorithm for computing the magnitude and gradients. At the final stage after computing the SVM score, they convert the fixed-point score value to floating-point and send it to the processor.

Luo et al. [11] propose a pure FPGA implementation of the HOG algorithm. They make several contributions which increase the frame rate of their design. For the bin assignment step, they use a comparison-based method instead of computing the arctangent. This method reduces hardware resource usage, but still their design requires four DSP (digital signal processing) cores for this part. They also propose an architecture for reusing the calculations in the block normalization step and dividing the SVM calculation into partial stages to decrease the overall latency.

Pure hardware implementations of the HOG algorithm have the advantage of a higher speed of calculations. Naturally, they consume more hardware resources than the work which assign parts of the tasks to a software processing system. There is a trade-off between the speed of the algorithm and resource utilization, which can be made based on the application and cost evaluation of the processing systems. In Section 3.2, we will review the work based on hardware–software co-design of the HOG algorithm.

### 3.2. Hardware–Software Implementation of the HOG Algorithm

In this work, we focus on the designs which propose a hardware–software co-design approach. The main advantage of these methods is that the resource usage of the hardware can be optimized while preserving the required speed for the application.

Mizuno et al. [18] propose a cell-based scanning scheme for implementing the HOG algorithm. They have parallelized modules for cell histogram generation, histogram normalization and SVM classification. Their proposed parallel architecture increases the speed while consuming more hardware resources. Their work is a hardware–software co-design, as they use CPU to control the pipeline of the HOG algorithm. They simplify the HOG computation by such methods as using the CORDIC algorithm for gradient calculation, using the Newton method for histogram normalization, and using specific bit-widths for different modules. They store the intermediate data of the histogram of the cells in SRAM memory and load the data for the further steps. Ma et al. [6] propose a hardware–software co-design approach for HOG-SVM computation. They profile the code on CPU to find the most critical and computationally extensive parts of the algorithm. As a result of their analysis, they implement histogram generation and block normalization on an FPGA. They store the result of block normalization in memory, and for the classification step, they re-load the normalized values from the memory. To minimize memory operations, they store the magnitude and orientation values of each single pixel as a 32-bit value in a single memory location. They propose a multi-scale design which computes HOG for 34 scales. They resize the image and compute the magnitude and gradient in software, and then store the result of this step in the memory on FPGA. In their design, the histogram generation and block normalization steps are assigned to the FPGA, and the results are written back to the memory. After that, the classification module loads the normalized histogram values from the memory and produces the final decision. They also use different bit-widths for different modules, similar to Mizuno et al. [18], so as to have a more efficient implementation. Rettkowski et al. [19] propose a hardware implementation, a software implementation, and a hardware–software co-design of the HOG algorithm. They implement their design on a Xilinx Zynq^®^ platform, and for their hardware–software model, they use a Linux operating system on the board. They also compute the histogram generation and block normalization steps on the FPGA. They use SDSoc^™^ software, which is an IDE (integrated development environment) by Xilinx for implementing heterogeneous embedded systems, to generate hardware modules, and due to the software limitations, they produce the results for 350 × 170 pixel windows in their hardware–software implementation. However, for their pure hardware implementation, they process 1980 × 1020 images and achieve a higher frame rate of 39.6 fps. Huang et al. [20] propose a hardware–software co-design of the algorithm by separating the classification and HOG computation parts. In their design, the HOG generation and computation is done on the FPGA, and the result is sent back to an ARM processor for the classification step. In order to improve classification, they use the Adaboost classifier first, followed by an SVM classifier to generate the final output. In the implementation by Ngo et al. [21], the classification step is done on software. They propose a sliding window architecture on hardware for the first part of the HOG algorithm. Bilal et al. [22] propose a simplification of the HOG algorithm by introducing a histogram of significant gradients. In their proposed method, only the gradients that have a value more than a threshold of average gradient magnitude of a block cast a binary vote to the histogram. Therefore, there is no need for a normalization step. They use HIK (histogram intersected kernel), which is a variation of the SVM as classification module, and implement it on a soft processor.

Existing hardware–software approaches have contributed significantly to the state-of-the-art, and research is ongoing to make further improvements. Some of the existing work, such as [18], requires multiple external memory accesses for intermediate results, which can lead to increasing the latency of the design. Another important observation in the existing work is that many include the processor in the flow of the data-path [6,19,20,21,23]. This can obviously become the bottleneck of the system, since the processor is usually slower than the programmable logic and processes data sequentially. In [20,21,22], the classification step is assigned to the software side of the system. Since classification is part of the data flow and can start as soon as the first block is processed, assigning it to the hardware part is a superior choice to increase the speed of the design. In this work, we propose a design which does not require any external memory access for computing an HOG descriptor for each window. We allocate the data-path of the algorithm to the programmable logic, and the control loops and address generation task to the processor. Therefore, the processor does not have negative impacts on the processing speed of the algorithm. In our design, we integrate feature extraction and classification in a unified pipeline to increase the speed of the process.

## 4. A Novel Hardware-Software Co-Design of the HOG-SVM System

In this section, we propose a hardware–software co-design system for HOG implementation. As a case study of the HOG algorithm’s application, we choose human detection, which is an online application. The INRIA person dataset [15] is one of the more commonly-used datasets for testing human detection approaches. In a real system, the input data would be captured using a digital image sensor, and then converted to grayscale, before being passed to the HOG feature extraction unit. For evaluation purpose, we use the image data from the INRIA dataset for training the SVM classifier and testing our implementation. We validate our design on a Xilinx^®^ FPGA (Kintex^®^ Ultrascale^™^) using Vivado^®^. Our contributions are made in two main ways. First are the algorithmic level enhancements, which are the new ideas inside the HOG-SVM core, including logarithm-based bin assignment, block normalization and parallel histogram computation. Second is at the task allocation level, which assigns the appropriate tasks to the processor system and programmable logic of the design.

In a human detection system, a frame of an image is considered as the input. We employ a sliding window technique, as in [5]. We use an 800 × 600 image resolution and a moving window size of 160 × 96 on the image. The frame size and the window size are based on the work by Luo et al. [11]. However, it could be readily changed for different applications. We extract the HOG features for all pixels and classify them using an SVM classifier. Since HOG feature extraction and classification are computationally expensive, we implement the HOG core in hardware in a fully pipelined manner. We allocate the image windowing step to software. This step is responsible for calculating the correct address of the image window in the memory and sending that address to the HOG core.

**Figure 4:**
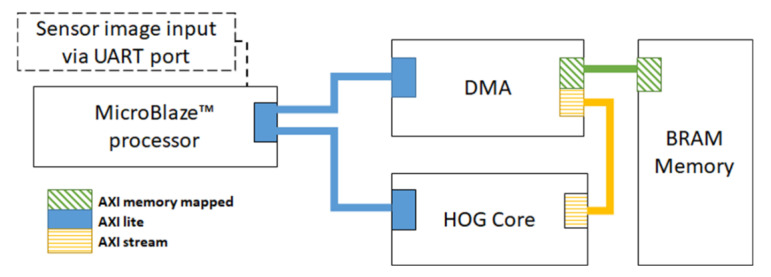
Block diagram of the proposed design and port connections.

The main parts of the proposed system are the MicroBlaze^™^ processor, a DMA (direct memory access) core and an HOG-SVM core. The MicroBlaze^™^ processor controls the main process by issuing the start signal to the HOG-SVM core and sending the address of an image to the DMA module. We assume that the input image is stored in the BRAM (block RAM) memory, which is the internal memory on the FPGA. This assumption is valid in multiple situations. There are many cases wherein other parts of a computer vision system acquire the image data and have loaded them beforehand in the BRAMs. In addition, since our primary focus is on the architecture of the HOG core, this assumption does not affect the main concept. We read the data from the BRAM in a raster scan streaming mode from the top left of the image to the bottom right. We divide each frame into several smaller windows, which can have overlaps with each other based on the required configuration. For each frame, the processor sends the address of the first pixel of the first row of a window to the DMA. The DMA, which is connected to the memory and the HOG core, reads one row of pixels from memory and sends that row to the HOG core in a streaming channel. The HOG core is designed using fixed-point numbers for efficiency. The core has two AMBA^®^ AXI interface ports. AXI is part of the ARM^®^ advanced microcontroller bus architecture, which provides a parallel high-performance interface. The first interface of the HOG core is based on the AXI light protocol, which is used for communications between the processor and HOG core. The second interface is an AXI stream protocol port which is connected to the DMA for high throughput data transfer. A simplified block diagram of the whole system is shown in Figure 4. We use the UART port as a matter of convenience to write the test image in the BRAM memory. Since the BRAM memory can be filled using various methods (depending on the application), this interface could be replaced with another connection interface without affecting the main concepts of this work.

When the DMA is moving data from the memory to the HOG core, one pixel is sent to the core at each clock cycle. There is a finite state machine inside the HOG core to control data receiving and processing. When new data are received, the core processes that data, and in the off times when the processor is sending the address of the next row (or the next window) to the DMA, the core enters a wait state. The whole system described in this section works with a maximum 150 MHz clock frequency. In the next section, we discuss the details of the HOG-SVM core.

## 5. HOG-SVM Core

**Figure 5:**
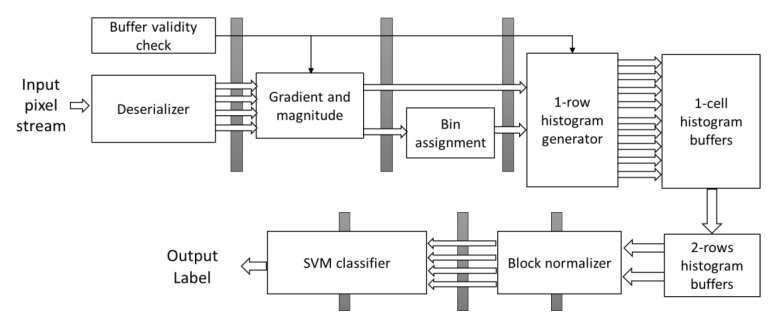
The overall diagram of the HOG-SVM core.

The overall diagram of the fully pipelined HOG-SVM implementation is shown in Figure 5. The solid gray bars represent the registers of the pipeline which we add to reduce the delay of the critical paths. The initial required time for filling the pipeline and generating the first input is 4.25 × W + 14 clock cycles, where W is the width of the image window. This initial setup time includes 3 × W clock cycles in the deserializer module, eight clock cycles in the one-row histogram generator module, W clock cycles in the one-cell histogram buffers module, W/4 clock cycles in the two-row histogram buffers module, and six clock cycles for the separation registers shown as gray solid bars in Figure 5, which are added to reduce the critical timing path of the combinational logic. Gradient and magnitude, and the bin assignment modules, are combinational. The deserializer, one-row histogram generator, one-cell histogram buffers, and two-row histogram buffers all have internal registers and are fully pipelined at the pixel level. When data reach the last stage of the core, all modules work in parallel and there is no need to stop or delay the streaming input in this pipeline. After that, at each clock cycle, one valid SVM output is generated. In this section, we describe the implementation details for each part and the novel contributions.

### 5.1. Deserializer and Buffer Validity Check

The first module of the HOG core is the deserializer unit. This module contains three line buffers which have the depth of the full image window. At every clock cycle, one pixel of the image is read and entered into the first register of the first line buffer, and the values of other registers are sent to the next adjacent registers. For the last register of the first row, the next register is the first register of the second row. Similarly, the value of the last register of the second row is sent to the first register of the third row. After reading three rows of the image, all three buffers are full, and then we can compute the gradients in horizontal and vertical directions.

**Figure 6:**
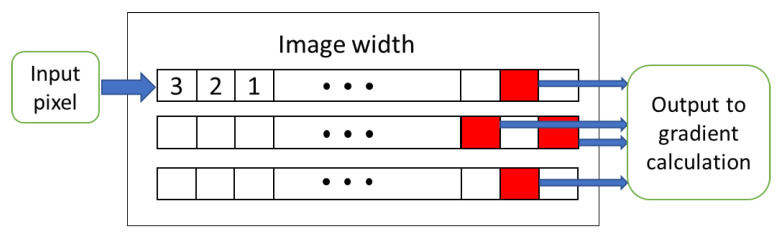
Line buffers in the deserializer module.

Figure 6 shows the buffers in the deserializer module. The red registers at the end of the line buffer contain the output pixel values of this module, which are sent to the gradient module. The numbers in the first row show the sequence of pixels entering the module. This module requires a setup time of 3 × W clock cycles to fill all registers before producing valid outputs.

The buffer validity check module is a set of counters which observe the input stream from the deserializer and issue control flow signals, to enable the gradient and magnitude calculation module and the histogram generator module. These signals are important in order to synchronize the flow of valid data in the pipeline.

### 5.2. Gradient and Magnitude Calculation

After deserializing the input stream, gradients in the horizontal and vertical directions are computed in the gradient and magnitude module. The gradient is computed using two subtraction units that subtract the right pixel from the left one and the top pixel from the bottom one. The magnitude of the gradient, which is approximated by the addition of the absolute values of gradients in horizontal and vertical directions, is obtained using two comparators and an adder unit. Since orientation computation and bin assignment are closely related to each other, we design a single unit for this step. Computed gradients are sent to this module for bin assignment.

As mentioned in [17], the original HOG algorithm requires 2 × W × H multiplication operations (for computing the square of the gradients twice for each pixel), W × H additions (once for each pixel), and W × H square root operations (once for each pixel) for computing the magnitude of gradients, where W is the width and H is the height of the image window. In our implementation, we simplified the magnitude computation by just performing W × H additions (for adding the absolute values once for each pixel) and 2 × W × H inversion operations (for absolute value of the gradients twice per pixel).

### 5.3. Logarithm-Based Bin Assignment

In this section, we introduce the new idea of logarithm-based bin assignment. The main advantage of this method is that there is no need to use multipliers, as in [10]. An embedded vision system could have multiple algorithms running simultaneously, and by not using multipliers we can save resources, such as DSP (digital signal processing) cores, for other parts of the system. The idea behind this design originates from the characteristic of logarithm function, which can be used to transform division into subtraction. Equations (7)–(10) demonstrate the mathematical procedure of this method. Equation (7) presents the original orientation computation comparison. In the logarithm-based method, we first compute the tangent of all values as in Equation (8). Then, we compute the absolute value and then the base 2 logarithm to all values, as in Equation (9). We do not lose any information by computing the absolute value, since we store the sign bit of $G_{y}$ for choosing the appropriate bin in the next step (we address this in detail later in this section). Subsequently, we separate the dividend and divisor of gradients, as shown in Equation (10). We compute the $\log_{2}\left(\left|\tan\left(\theta_{i}\right)\right|\right)$ offline, and just calculate the $\log_{2}\left(\left|G_{x}\right|\right)$ and $\log_{2}\left(\left|G_{y}\right|\right)$ values on the FPGA.

(7) $$\theta_{i}<tan^{-1}\left(\frac{G_{y}}{G_{x}}\right)\leq \theta_{i+1}$$

(8) $$\tan(\theta_{i})<\frac{G_{y}}{G_{x}}\leq \tan(\theta_{i+1})$$

(9) $$\log_{2}\left(\left|\tan(\theta_{i})\right|\right)<\log_{2}\left(\left|\frac{G_{y}}{G_{x}}\right|\right)\leq \log_{2}\left(\left|\tan\left(\theta_{i+1}\right)\right|\right)$$

(10) $$\log_{2}\left(\left|\tan(\theta_{i})\right|\right)<\log_{2}\left(\left|G_{y}\right|\right)-\log_{2}\left(\left|G_{x}\right|\right)\leq \log_{2}\left(\left|\tan\left(\theta_{i+1}\right)\right|\right)$$

**Figure 7:**
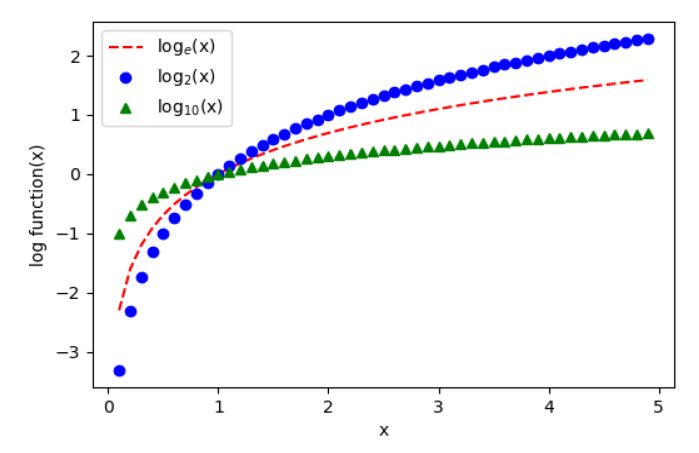
Difference between the slope of three logarithm functions.

The reason that $\log_{2}$ is chosen in this method is because of the bigger slope that this function has in comparison with $\log_{10}\;$or $\log_{e}$. Figure 7 shows the difference in slopes among these functions. The greater the slope of the function is, the more differentiable the output is. By using the function with a greater slope, the precomputed logarithm values can then be scaled with a smaller ratio, thus minimizing quantization errors.

To compute the $\log_{2}$ values, we use an LUT-based (Look Up Table) RAM. Depending on the input value which is the computed gradient, we can choose the appropriate $\log_{2}$ values, which are stored in the LUTs of the FPGA.

After retrieving the $\log_{2}\;$values, we subtract $\log_{2}\left(\left|G_{y}\right|\right)$ and $\log_{2}\left(\left|G_{x}\right|\right)$ from each other, and we find the appropriate bin based on the subtraction result. To make our design more accurate by taking into account the hardware resource restrictions, we scale the values so that we can prevent mantissa numbers. Equation (11) shows the scaled version of (10). Both sides of the inequality are precomputed, and for the middle expression, one addition to 160 is added. The reason for adding 160 is that we multiply all sides of (8) by 32, and then compute the $\log_{2}$ of them. Then, we multiply the logarithm values by 32. Since ${32\log}_{2}\left(32\right)$ is equal to 160, we add 160 to the middle expression. The LUT-based $\log_{2}$ is to calculate ${32\log}_{2}$ instead of log_2_, and only the absolute values of $G_{x}$ and $G_{y}\;$are given to these LUTs as input.

(11) $$32\log_{2}\left(32\left|tan(\theta_{i}\right|\right)<160+32\log_{2}\left(\left|G_{y}\right|\right)-32\log_{2}\left(\left|G_{x}\right|\right)\leq 32\log_{2}\left(32\left|tan\left(\theta_{i+1}\right)\right|\right)$$

**Figure 8:**
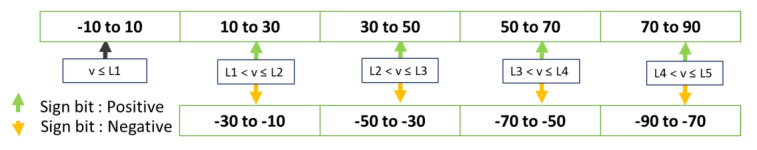
The bin assignment procedure.

After that, the appropriate bin is selected using the sign bit, as in Figure 8. In this figure, L1 to L5 represent precomputed limits for deciding the appropriate bin. After the range of the number is determined, the appropriate bin is selected according to the sign value. In Figure 8, v is the term computed by subtraction of the logarithm values. Depending on the sign bit, a range of the orientations is chosen.

The limit values in Figure 8 are shown in Table 1. These limits are precomputed values of ${32\log}_{2}\left(\tan\left(\theta_{i}\right)\right)$, where $\theta_{i}$ is the bin limit between −90 and 90 degrees.

The pseudo-code for the bin assignment step is shown in Algorithm 1.
**Algorithm 1** The pseudo-code for the bin assignment stepCalculate the absolute values of $G_{y}$ and $G_{x}$Store the sign of ($G_{y}$)×($G_{x}$) in the sign bitCalculate the scaled logarithms of $G_{y}$ and $G_{x}$Based on the log value, map to the −90 to 0 degrees bins if the sign bit is negativeBased on the log value, map to the 0 to +90 degrees bins if the sign bit is positive

It is important to note that for this part, each bin is represented with 12 bits to maintain the accuracy. As mentioned in [17], in the original HOG algorithm, the orientation and bin assignment module require W × H arctangent operations, W × H divisions, and 9 × W × H comparison operations. Although some previous works [10] use 18 × W × H multiplication operations instead of arctangent, by using our method, the bin assignment module does not use any multipliers. It computes the appropriate bin only by using W × H subtractions (for the $\log_{2}\left(\left|G_{y}\right|\right)$ and $\log_{2}\left(\left|G_{x}\right|\right)$ subtraction), 9 × W × H comparisons (for bin assignment) and 2 × W × H inversions (for the absolute value of gradients), and reading values from LUTs. As a result, multipliers and DSP units are saved for other possible processes required in the vision system.

### 5.4. One-Row Histogram Generator

**Figure 9:**
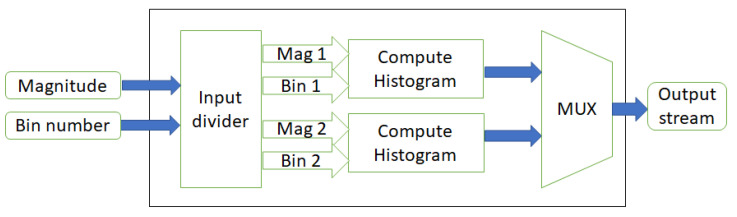
One-row histogram generation module.

**Figure 10:**
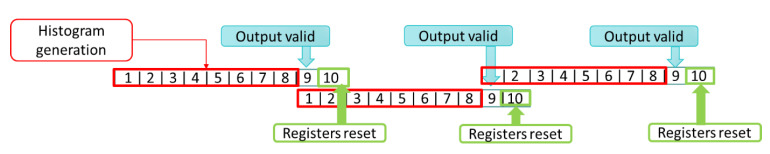
The time-sharing protocol for the histogram generation module.

We describe the implementation of a one-row histogram generator unit in this section. This module gets the magnitude and bin assignment inputs from the previous modules. Then, according to the orientation related to each magnitude, a histogram is created for every eight pixels. Computing the histogram requires more than eight clock cycles. This module contains nine registers representing each bin. In the first eight clock cycles, the input enters this module, and the value of each bin is added to the appropriate register, representing an orientation bin. This module requires one clock cycle to output the completed partial histogram, and one clock cycle to reset the registers to zero again to become ready for the next incoming pixels. Since computing histograms in this way requires the input data stream to pause, we design this step by using two partial histogram generators, which work in parallel using a time-sharing protocol. As illustrated in Figure 9, the input divider sends a valid magnitude and bin number to the compute histogram modules, and the multiplexer at the end chooses the valid histogram based on the time-sharing protocol. Figure 10 demonstrates how the time-sharing protocol works for each eight pixels entering the one-row histogram generator module. Each module requires eight clock cycles to create the histogram, one clock cycle to put it on the output port and one clock cycle to reset the registers. At the 9th clock cycle the output is valid, and at the 10th clock cycle, we reset the registers. While one of the compute histogram modules is in output and reset phase, the other one gets the input stream of data and continues the process. Therefore, there is no need to stop the streaming input. Otherwise, we should pause the streaming input for one cycle for each cell calculation, which could slow a design, especially when processing high-resolution frames.

### 5.5. One-Cell Histogram Buffers

We employ the same architecture proposed by Luo et al. [11] for designing one-cell histogram buffers and two-row histogram buffers. The one-cell histogram buffers module computes the histograms of each eight pixels in a row. The output of this module is nine 16-bit bins of eight pixels in a row every clock cycle. Since our goal is to compute the histogram for 8 × 8 cells, we use histogram buffers to store the computed histograms sent from the one-row histogram generator module.

This module contains two parts. The first part has eight lines of buffers. Each line has eight buffers. At each clock cycle, a histogram of eight pixels enters this module into the first line buffer. Then, the line buffers work as a shift register, and at each clock cycle, the values are moved through the line buffers. When the first entry of the line buffers reaches the last register, the data in the last register of each line are the histograms of eight pixels of each row of a cell. Therefore, by adding them together bin by bin, we can derive the histogram of a cell. On the next clock cycle, the histogram of the next cell is computed. This process continues until the cell line in the image is changed. While the line buffers are loading up again, their output is not valid.

**Figure 11:**
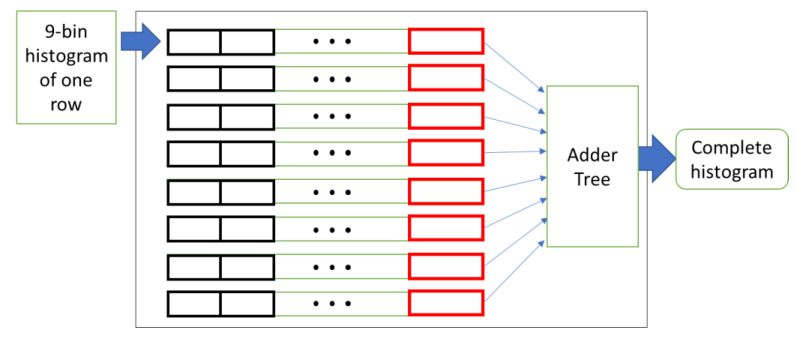
The block diagram of one-cell histogram buffers.

Figure 11 illustrates the eight line buffers of this module. We use a tree-based adding structure to minimize the critical path of the combinational logic for addition. Since we have eight arguments from eight buffers, the tree-based adding structure will have three levels. Therefore, by using a three-level tree-based adding structure, the histogram of a cell can be computed efficiently. This module requires W/8 clock cycles to fill the first row of the buffers, since the input of this module is a histogram computed for eight pixels. Since there are eight rows in this module, a total number of W clock cycles is required to fill the buffers of this module and generate the first valid output.

### 5.6. Two-Row Histogram Buffers

**Figure 12:**
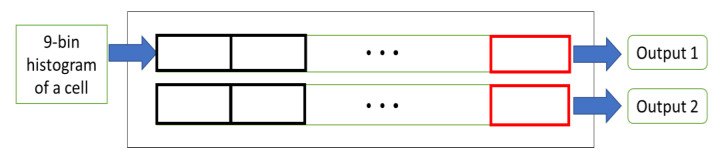
The block diagram of two-row histogram buffers.

The next stage is the two-row histogram buffers. The objective of this module is to deserialize the computed cells to have access to four adjacent cells in parallel. Figure 12 shows the block diagram of this module. At each clock cycle, if the input is valid, a nine-bin histogram enters these line buffers. When the first cell which has entered this module reaches the last register, we have the histograms of the cells of two cell rows ready at the same time. These values are the output of this module. This module requires a setup time of 2 × W/8 clock cycles to generate the first valid output, since there are two rows and we have W/8 registers in each row.

### 5.7. Block Normalization

The next stage of this design is block normalization. For accurate implementation, if we want to have a latency of one clock cycle for the normalization, 36 multipliers, one square root operation and one division are required. The other possible design is to use one multiplier and compute the square operation once in each clock cycle, which will add 36 clock cycles for the normalization of each block. In this work, we propose a simplified design for the block normalization step. Our design normalizes each histogram bin so that the summation of all bins in a block is less than a specific threshold. Choosing a larger value for this threshold will result in less approximation and therefore more accuracy. However, it will consume more hardware resources, since we must dedicate more bits to the result. We choose 255 for this limit as a trade-off between accuracy and resource usage. In addition, we use division by powers of two, which simply shifts the input value and is much less resource-consuming than other division algorithms.

**Figure 13:**
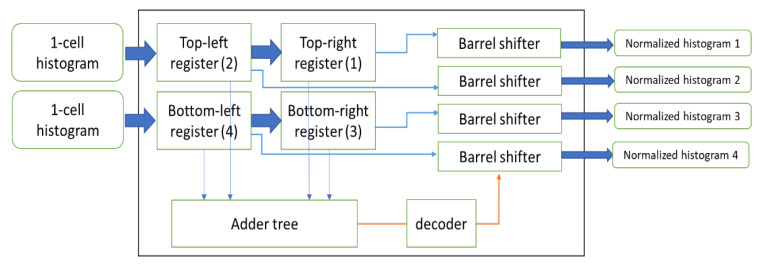
The block diagram of block normalization module.

Figure 13 shows the block diagram of the normalization module. The block normalization module receives two histograms from two cells in one cell column at each clock cycle, and stores them in the top-left and bottom-left registers. Since the normalization is done for every four cells, this module stores the two inputs for a clock cycle in the top-right and bottom-right registers. In the subsequent clock cycle, when all four histograms are ready, the block normalization module computes the normalized value. First, all bins of the four histograms are added to each other using a tree-based adding structure. Then, depending on the four most significant bits of the sum value, a step-based normalization is adapted using a decoder. Then, each histogram is shifted using a barrel shifter.

We aim to limit the summation of each block to 255, as shown in Table 2. Therefore, depending on the location of the most significant bit that is a ‘1’ in the sum value, all histogram values are divided. If the value of the summation is more than 2047, we divide all histograms by 16. If it is less than that, depending on the bit number, we shift the histograms to the right (each shift divides by two) in order to keep the summation in the range of 0 to 255.

We can benefit from checking one bit of the summation by using binary values for comparison and division. We also perform division by shifting the histogram values, and therefore avoid a complex divider circuit. As mentioned in [17] in the original HOG algorithm, the block normalization step requires 9 × C multiplication (for the square of each histogram bin), addition and division operations, and B square root operations, where C is the total number of cells and B is the total number of blocks in an image window. Our simplification results in having 35 × B addition operations (for the adder tree) and 36 × B shifting operations (for four cells in each block).

### 5.8. SVM Classifier

**Figure 14:**
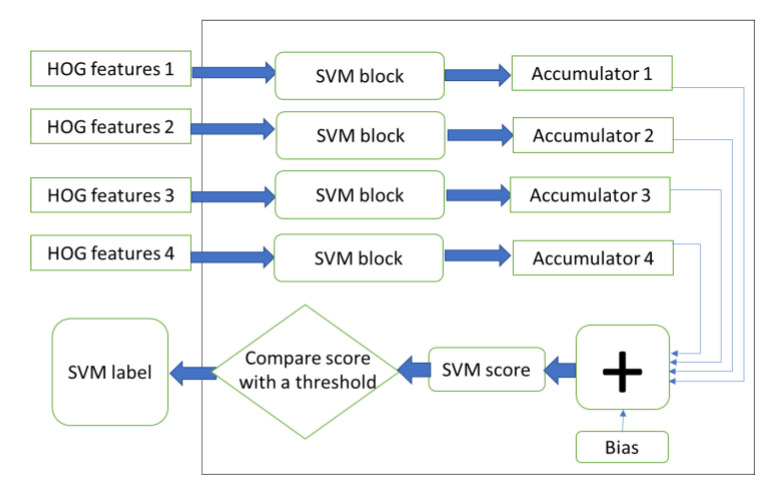
The SVM classifier module.

The last part of the HOG-SVM core is the SVM classifier. In this stage, the output of the block normalization step is given as an input. Since four histograms are normalized at each clock cycle, the SVM module gets four nine-bin histograms as input at once. These histograms are given to the four SVM blocks in this module, as shown in Figure 14.

**Figure 15:**
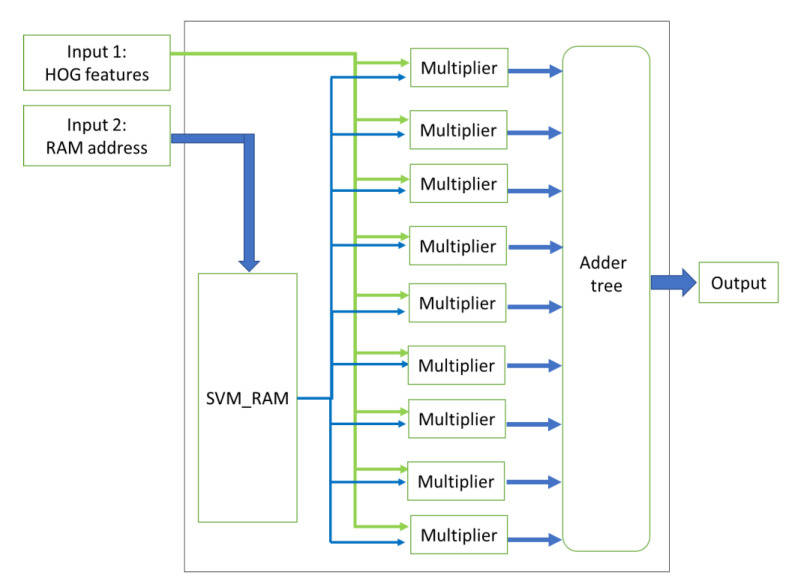
SVM block internal logic.

Each of the four parallel SVM blocks contains an SVM RAM, which holds precomputed weights for the SVM classifier. In each SVM block, the input histograms are multiplied bin by bin to the trained weights of the SVM classifier, and their results are added together in four accumulators. The internal logic of an SVM block is illustrated in Figure 15. This unit contains an SVM RAM which has the precomputed weights. Nine multipliers are working in parallel in each SVM block module. Finally, when all the data are processed, the values of the accumulators and the bias term of the SVM classifier are added, which is the final score of the SVM classifier. By comparing this score with a predefined threshold, the SVM will indicate if the image window is a positive or negative sample. If the score is more than the threshold, the label is one, and otherwise, it is zero. In terms of the number of operations, the SVM classifier module requires B comparisons, 36 × B multiplications and 40 × B addition operations, where B is the total number of blocks in an image window.

## 6. Results and Comparison with Other Work

Rettkowski et al. [19] were among the first to demonstrate the speed gain of a pure hardware implementation of the HOG algorithm over a software implementation. Pure hardware implementation consumes more resources than when some part of the computation is done on the processor. However, computational approximations in hardware implementations can lead to some accuracy loss. The hardware–software co-design provides a trade-off between preserving the accuracy and limiting hardware resource usage. Therefore, such a design should be compared to other hardware–software designs which are facing the same trade-off in order to have a fair comparison.

Unlike most previous work [6,19,20,21,22,23], in our design, the flow of the data does not include the processor itself. This is important since in those cases, the processor would be the bottleneck of the system. In addition, the HOG-SVM core is designed so that no memory access is required for intermediate computations, as in [18]. Intermediate communications with external off-chip memory reduce the speed of the system. In our design, everything is buffered using on-chip FPGA resources. Another advantage of our design is that when the first block of the normalized histograms is ready, the classification step starts, and at each clock cycle, one block of data is given to the classifier. Classification is part of the data flow, and assigning it to software as in [22] could decrease performance. At the algorithmic level, we make three contributions. By using the logarithm-based bin assignment, we save four multipliers (DSP units), which can be used for other possible computations or applications on the same chip. By using a simplified block normalizer, we save 36 multipliers, one division unit and one square root operation. In addition, by employing parallel histogram computation, we save 20% of the time for each histogram’s generation. We provide the results of our implementation and comparison with other work in Table 3. The numbers provided by other work in this table are obtained from their published results.

The last column of Table 3 demonstrates the metric of pixel per clock cycle. Our proposed design has a larger pixel per clock cycle value than most of the other hardware–software methods. The work by Long et al. [13] has the highest pixel per clock cycle value. The reason is that in [13], the input of the system is 64 pixels per clock cycle, while others receive one pixel per clock cycle as an input. The work by Mizuno et al. [18], which achieves the highest pixel per clock cycle value in the hardware–software co-design work (due to their highly parallel architecture), uses about twice the number of DSPs and about four times more LUT resources than our proposed design for the same image resolution. In that sense, our design is more efficient in the case of resource usage and, after the initial setup time, can produce a valid output at each clock cycle. Pure hardware implementations are typically faster than hardware–software implementations. However, they will often require more hardware resources as a trade-off.

As shown in Table 3, Rettkowski et al. [19] use a Zynq^®^ family FPGA, while Ma et al. [6] use a Virtex^®^ series FPGA. Mizuno et al. [18], Bilal et al. [22] and Ngo et al. [21] use Cyclone^®^ family FPGAs. Cyclone^®^ V devices have more available memory, while Virtex^®^ family FPGAs have more logic elements than Cyclone^®^ and Zynq^®^ series. The latest FPGAs and technologies should lead to faster systems, however innovative implementation is also a big driving factor in making an effective and efficient system. Ma et al. [6] implement HOG in 34 scales but use the FPGA resources more extensively than other work.

**Figure 16:**
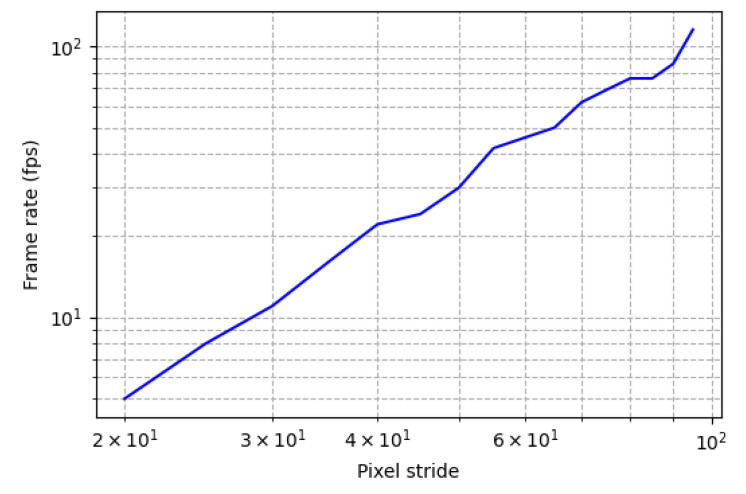
The relation between pixel stride and frame rate.

The results of Table 3 indicate that our system uses a comparable number of DSPs and BRAMs in processing images of similar size, and fewer LUT resources than other work which implement hardware–software co-design systems and pure hardware systems. The frame rate mentioned in Table 3 is for the case in which there is no overlap between sliding windows. If we increase the number of overlapped pixels (or decrease pixels stride) the frame rate decreases. Stride is the number of pixels between the current window and the next window in one direction. Figure 16 demonstrates the relationship between frame rate and pixel stride in a logarithmic scale. This figure shows that there is a near linear relationship between frame rate and pixel stride.

Since the sliding window part of the system only has the responsibility of calculating the correct address of the windows, it is reasonable to choose the processor for this task. On the other hand, HOG and SVM calculations, which require many additions, multiplications and comparisons, are more efficient using hardware. Our design is well-suited for applications, such as mobile and embedded systems, where there is a limitation in hardware resources. By minimizing the usage of hardware resources by HOG and SVM, there are more resources available for other parts of an application, and we can still get accurate and comparable results. Table 4 illustrates the resource usage of all parts of the HOG-SVM IP-core.

We present the resource usage of the whole system in Table 5. The reset and clock module is responsible for creating the required clock frequencies, and distributing clock and reset signals to all parts of the design. MicroBlaze^™^ is the main processor, which contains local memory, a debug module, a peripheral controller and an interrupt controller. We use AXI Data FIFO to buffer the streaming information from the DMA module to the HOG-SVM IP-core.

To measure the speed of the design, we load the input image into the BRAM memory of the FPGA. In our experiments we use 800 × 600 images so as to be comparable with other hardware–software co-design work, since published results are mostly at this resolution. However, using a higher image resolution such as 1920 × 1080 does not affect our implementation in terms of resource usage, since the required resources are based on the image window size and not the whole image. The processor starts the computation by instructing the DMA to read from the memory and send the data to the HOG-SVM IP-core. Since in a practical application an external memory can be used and the image can have any arbitrary size, we did not report the number of BRAM memories dedicated to the image stored on the FPGA in Table 5, as it is not one of the main elements of the proposed system.

The bandwidth of the designed streaming channel between the memory and the HOG-SVM IP-core is 1.2 Gbit/s, since the DMA can send each pixel in one clock cycle to the HOG core. In our design, for each line of the image, the processor sends a command to DMA to start the data transfer for a specific number of pixels. Although this controlling mechanism gives the system the capability to process different sizes of the image, it adds an overhead to the timing. Therefore, the data rate of the transfer between the memory and the HOG-SVM IP-core is decreased to 55 Mbit/s, based on our measurements.

In terms of the number of operations, as mentioned in detail in Section 5, our proposed design has reduced the W × H + 9 × C additions, 2 × W × H + 9 × C multiplications, W × H arctangent operations, W × H + 9 × C divisions and W × H + B square root operations in the original HOG algorithm to 2 × W × H + 35 × B additions, 4 × W × H inversions, 9 × W × H comparisons and 36 × B shifting operations, where C is the total number of cells in an image window. These numbers exclude the parts which were similar, such as the operations required by the SVM module. The SVM module requires B comparisons, 36 × B multiplications and 40 × B addition operations, where B is the total number of blocks in an image window.

**Figure 17:**
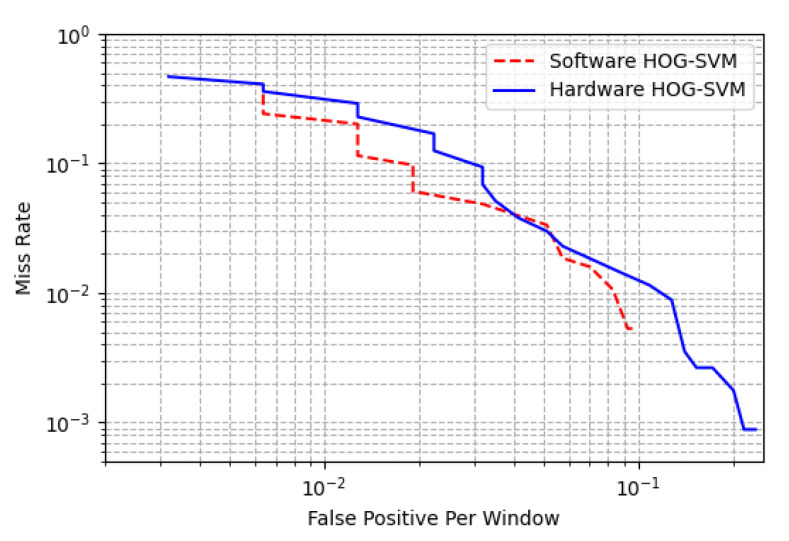
Comparison of software implementation and our proposed HW-SW co-design.

We used a hardware model in MATLAB^®^ for evaluating the accuracy of the design. The hardware model produces identical results to the actual implementation on the FPGA. This procedure is similar to the work by Luo et al. [11]. The accuracy results of our system are shown in Figure 17. It can be observed that for the test set of the INRIA dataset, the accuracy of our design is very close to, but slightly lower than, that of the software implementation of the algorithm, which is due to the quantization of the floating-point values and simplifications in hardware. Figure 17 demonstrates miss rate versus false positive per window, which is the most common method for evaluating human detection systems. The vertical axis shows the miss rate and the horizontal axis represents the number of false positives per window. This diagram is typically drawn in a log–log scale. The software version is our implementation of the HOG-SVM, using MATLAB^®^ software and the Statistics and Machine Learning Toolbox based on [5].

## 7. Conclusions

In this work, we proposed a hardware–software co-design system of the HOG algorithm, which can receive input data from a digital image sensor, extract the HOG features and make a decision based on those features. Our implementation makes four main contributions. First, at the task allocation level, we propose a well-organized partitioning between different parts in a hardware–software co-design system, which consumes fewer FPGA resources than other comparable hardware–software systems. The idea is to assign the computationally intensive parts of the algorithm, such as gradient and magnitude computation, bin assignment, normalization and classification, to hardware, and delegate the resource-intensive part, which is the windowing stage, to software. Second, as an algorithmic-level contribution, to the best of our knowledge, we are the first to propose a logarithm-based bin assignment in the HOG algorithm, which leads to a multiplier-free implementation of the HOG and reduces the overall number of multipliers for the HOG-SVM core. Third, we propose to use two parallel histogram computation modules, which save one clock cycle for every 8 pixels. As a result, the HOG core can accommodate the pixel data in a streaming manner on each clock cycle without any pause. Finally, we propose a simpler implementation of the block normalization step, which reduces the IP-core resources.

Our design has the capability to use several HOG-SVM IP-cores in parallel for one image. In future, we can modify the design to take advantage of this feature and enhance the speed of the system. Another possibility is to use interrupts efficiently to read precomputed window addresses from the memory. In this way, the processor would have more free time to perform other tasks while the HOG-SVM cores and DMAs are processing the image. Another possible enhancement involves developing other variants of the HOG algorithm and their implementation in hardware. There are many other variants of the HOG algorithm, such as HOG-3d [24], which require a high number of computations and can benefit from parallel implementation.

## Figures and Tables

**Table 1:** Limits for bin assignment.

Limits	Value
L1	80
L2	135
L3	168
L4	207
L5	347

**Table 2:** Block normalization decoding method.

Limits of Values for Sum	Bits of the Summation	Division of All Histogram Bins	Number of Bits to Shift the Histograms
Sum > 2047	Bit 11 is checked	Histogram/16	Histogram >> 4
2048 > Sum > 1023	Bit 10 is checked	Histogram/8	Histogram >> 3
1024 > Sum > 511	Bit 9 is checked	Histogram/4	Histogram >> 2
512 > Sum > 255	Bit 8 is checked	Histogram/2	Histogram >> 1
Sum < 256	---	Histogram	Histogram

By checking the most significant bits of summation one after another, we can find the range of sum. Based on that, we divide all histograms by shifting the bits to right.

**Table 3:** Comparison with other work.

	Reference	FPGA	Image Size	LUTs	BRAM (Kbit)	DSP	Frame Rate (fps)	Pixel per Clock Cycle
Pure Hardware Design	Rettkowski et al. [19] ^1^	Zynq^®^	1920 × 1080	41,858	1584	13	39.6	0.99
	Ngo et al. [14]	Cyclone^®^ V	640 × 480	13,646	317	38	75	0.46
	Long et al. [13]	Stratix^®^ IV	512 × 512	266,023	47	236	2500	8.19
	Luo et al. [11]	Cyclone^®^ IV	800 × 600	16,060	334	69	162	0.51
	Qasaimeh et al. [12]	Zynq^®^	1920 × 1080	32,871	NA	130	48	0.59
Hardware–Software Co-design	Mizuno et al. [18]	Cyclone^®^ IV	800 × 600	34,403	334	68	72	0.86
	Ma et al. [6]	Virtex^®^-6	640 × 480	184,953	13737	190	68	0.14
	Bilal et al. [22]	Cyclone^®^ IV	640 × 480	65,501	103	10	25	0.15
	Yu et al. [23]	Spartan^®^-6	640 × 480	15,167	351	19	1.5	NA
	Rettkowski et al. [19] ^1^	Zynq^®^	350 × 175	NA	NA	NA	0.44	0.0001
	Ngo et al. [21]	Cyclone^®^ V	640 × 480	12,138	437	65	11	0.02
	Hunag et al. [20]	Spartan^®^-6	384 × 288	NA	NA	NA	25	NA
	Our HW-SW co-design	Kintex^®^ UltraScale^™^	800 × 600	7804	756	36	115	0.37

^1^ This work did not implement an SVM.

**Table 4:** HOG-SVM IP-core resources.

Module Name	LUTs	Block RAM Tile	DSP
De-serializer	117	0	0
Buffer validity check	62	0	0
Gradient and Magnitude	8	0	0
One-row histogram generator	502	0	0
One-cell histogram buffers	1960	0	0
Two-row histogram buffers	376	0	0
Block normalizer	799	0	0
SVM classifier	1622	0	36
Overall	5658	0	36
Percentage used ^1^	1.06%	0	1.87%

^1^ The percentage value is based on the FPGA resources of the KCU105 FPGA board used in this work.

**Table 5:** Resource usage of the whole hardware–software system.

Module Name	LUT	Block Ram Tile (36Kbit)	DSP
Reset and Clock	17	0	0
Microblaze^™^	1114	0	0
Microblaze^™^ local memory	11	16	0
Microblaze^™^ Debug Module	156	0	0
Microblaze^™^ Peripheral Controller	179	0	0
Microblaze^™^ Interrupt Controller	69	0	0
AXI Data FIFO	56	0.5	0
DMA	544	4.5	0
HOG-SVM core	5658	0	36
Sum	7804	21	36
Percentage used ^1^	1.47%	3.5%	1.87%

^1^ The percentage value is based on the FPGA resources of KCU105 FPGA board used in this work.
